# Privacy and Confidentiality Concerns Related to the Use of mHealth Apps for HIV Prevention Efforts Among Malaysian Men Who Have Sex With Men: Cross-sectional Survey Study

**DOI:** 10.2196/28311

**Published:** 2021-12-16

**Authors:** Roman Shrestha, Celia Fisher, Jeffrey A Wickersham, Antoine Khati, Rayne Kim, Iskandar Azwa, Colleen Mistler, Lloyd Goldsamt

**Affiliations:** 1 Department of Allied Health Sciences University of Connecticut Storrs, CT United States; 2 Center for Ethics Education Fordham University New York, NY United States; 3 Department of Internal Medicine Yale University New Haven, CT United States; 4 Department of Medicine University of Malaya Kuala Lumpur Malaysia; 5 Rory Meyers School of Nursing New York University New York, NY United States

**Keywords:** HIV, men who have sex with men, mHealth, ethics, Malaysia, mobile phone

## Abstract

**Background:**

The use of mobile health (mHealth), including smartphone apps, can improve the HIV prevention cascade for key populations such as men who have sex with men (MSM). In Malaysia, where stigma and discrimination toward MSM are high, the mHealth platform has the potential to open new frontiers for HIV prevention efforts. However, little guidance is available to inform researchers about privacy and confidentiality concerns unique to the development and implementation of app-based HIV prevention programs.

**Objective:**

Given the lack of empirical data in this area, we aim to understand the privacy and confidentiality concerns associated with participation in a hypothetical app-based research study for HIV prevention efforts.

**Methods:**

A cross-sectional, web-based survey was conducted between June and July 2020 among 355 Malaysian MSM. The survey included demographic and sexual health questions and a series of short videos describing a hypothetical app-based HIV prevention program, followed by questions related to privacy and confidentiality concerns in each step of the app-based program (ie, recruitment, clinical interaction, risk assessment, and weekly reminder). Multivariable logistic regression models were used to identify the correlates of willingness to use such an app-based program.

**Results:**

Most of the participants (266/355, 74.9%) indicated their willingness to participate in a hypothetical mHealth app–based HIV prevention program. Participants expressed concerns about privacy, confidentiality, data security, and risks and benefits of participating in all stages of the app-based HIV research process. Multivariate analyses indicated that participants who had a higher degree of perceived participation benefits (adjusted odds ratio [aOR] 1.873; 95% CI 1.274-2.755; *P*=.001) were more willing to participate. In contrast, participants who had increased concerns about app-based clinical interaction and e-prescription (aOR 0.610; 95% CI 0.445-0.838; *P*=.002) and those who had a higher degree of perceived risks of participating (aOR 0.731; 95% CI 0.594-0.899; *P*=.003) were less willing to participate.

**Conclusions:**

Overall, our results indicate that mHealth app–based HIV prevention programs are acceptable for future research on Malaysian MSM. The findings further highlighted the role of privacy and confidentiality, as well as the associated risks and benefits associated with participation in such a program. Given the ever-evolving nature of such technological platforms and the complex ethical–legal landscape, such platforms must be safe and secure to ensure widespread public trust and uptake.

## Introduction

### Background

HIV transmission among men who have sex with men (MSM) continues to accelerate and fuel the global epidemic, especially when stigma and discrimination are high [1,2]. Malaysia, a middle-income country with low (0.4%) overall HIV prevalence, has an evolving epidemic in MSM, with new HIV cases attributable to MSM increasing from 10% in 2008 to 54% in 2016 [3]. Biobehavioral surveys suggest that HIV prevalence among MSM is now at an all-time high of 21.6%. The highest prevalence is concentrated in the country’s capital, Kuala Lumpur, where HIV prevalence among MSM was 43% in 2018, up from 22% in 2014 [4]. Central to the expanding HIV epidemic among Malaysian MSM is the concomitant use of drugs, especially amphetamine-type stimulants, linked to condomless sex and associated sexually transmitted infections [5-7]. In Malaysia, where both homosexuality and drug use are criminalized, MSM who use drugs bear the burden of social stigma and discrimination. Consequently, they are marginalized from traditional, venue-based HIV prevention approaches and require innovative HIV prevention strategies that improve coverage and access [8,9].

In the past decade, mobile phone technology has become increasingly ubiquitous and has allowed the use of new approaches, including mobile health (mHealth), to improve health outcomes [10-13]. mHealth tools can deliver effective HIV prevention in a confidential, less-stigmatizing, and convenient manner—all features that are crucial for prevention efforts among stigmatized and hard-to-reach populations, such as Malaysian MSM. MSM in Malaysia often do not access HIV prevention services, given the existing and limited service delivery models for the lesbian, gay, bisexual, and transgender (LGBT) community [14]. Recent studies with Malaysian MSM have shown that nearly all (>97%) of them own a smartphone. Many have stated preferences for interacting with smartphone apps to access HIV prevention services to avoid risk and shame because of disclosure [15,16]. As such, the use of HIV prevention apps holds promise for reducing transmission among this at-risk group [17-23], especially when linked to effective biomedical preventions, including pre-exposure prophylaxis [8,9,24]. However, no smartphone-based app tailored to the specific needs of MSM in Malaysia has been developed to improve access to HIV prevention services for this at-risk group.

Several studies have evaluated the efficacy of mHealth-based HIV prevention interventions targeting MSM populations globally [24-26]. mHealth is a trending research topic in Malaysia, as the number of published studies on the interest and use of mHealth technologies has exponentially increased over the past 10 years [27]. However, privacy and confidentiality risks remain to be primary concerns for sexual and gender minorities participating in mHealth research studies [28-35]. Privacy concerns around collecting sensitive information (eg, sexual and drug use behaviors) may limit willingness to use and engage in an HIV prevention mHealth app for MSM in Malaysia [28,36-38]. To date, no studies have examined whether any of these factors influence the decision among MSM to participate in an mHealth app–based HIV prevention program in Malaysia.

### Objectives

Understanding the motivations of MSM to participate in mHealth studies and the perceived privacy concerns is essential for increasing their representation in research designed to empirically validate prevention strategies. Given the lack of empirical data in this area of mHealth research and an existing mHealth app for HIV prevention in Malaysia, we aim to understand the perceived privacy and confidentiality issues associated with using an mHealth app for HIV prevention efforts among MSM in Malaysia. Specifically, this exploratory study evaluates the motivation of MSM in Malaysia to participate in a hypothetical mHealth app–based HIV prevention program. These data are crucial to developing an ethically sound, app-based HIV prevention program while minimizing any unintended harm, such as third-party use, incidental discovery by someone, or legal interception.

## Methods

### Study Design and Participants

A cross-sectional, web-based survey of Malaysian MSM was conducted from June to July 2020 to assess their attitudes toward participation in a hypothetical mHealth app–based HIV prevention program. Individuals were eligible if they (1) were ≥18 years; (2) identified as male; (3) had a self-reported HIV status as negative or unknown; (4) reported substance use or condomless anal sex with another man in the past 6 months; and (5) were able to read and understand English or Bahasa Malaysia.

### Study Procedures

A sample of MSM was recruited through purposive sampling via advertisements in 2 venues: geosocial networking (GSN) apps for MSM (eg, Grindr and Hornet) and popular social networking websites for the general population (eg, Facebook). The GSN apps pushed the advertisement as a message to the chat inboxes of all their different users in Malaysia. On the social networking site, we posted flyers on nongovernmental organizations and community-based organizations that provide services to MSM. In addition, we used banner advertisements that were targeted at MSM aged ≥18 years residing in Malaysia. These targeted advertisements could appear in one of two ways: a static advertisement on the right-hand pane of the website or an advertisement that resembled a standard post as users scrolled through their feeds. Interested users who clicked on advertisements were directed to an eligibility screening tool and a brief web-based consent form hosted by Qualtrics. The consent form indicated that they could “enter a lucky draw to win 1 of 10 RM 635 vouchers” (equivalent to US $150) and that there was “no participation necessary” to enter the random drawing.

Eligible volunteers completed a web-based consent form acknowledging that they understood the study’s purpose, risks, and benefits before completing the survey. Those who declined to participate were provided instructions on how they could enter the drawing. Participants who completed the survey and were interested in entering the drawing were redirected to a separate website in which they entered an email address that was not linked to their data. On average, the participants took 10-12 minutes to complete the anonymous web-based survey. The study protocol and consent form were approved by the University of Malaya Research Ethics Committee and the Yale University Institutional Review Board.

We followed a protocol based on published standards for removing potentially duplicate cases while erring on the side of keeping rather than removing data in cases where a determination could not be made [39]. In particular, we identified potential duplicates based on age, sexual orientation, and ethnicity. All cases sharing those features in common were manually examined, focusing on responses to other questions, such as education, relationship status, income, device and browser information, and survey duration.

### Study Measures

#### Participant Characteristics

These included age, sexual orientation, ethnicity, relationship status, educational attainment, income, self-reported history of HIV testing practices, disclosure of sexual orientation to others, and perceived stigma and discrimination from health care providers.

#### Smartphone Access and Use

Participants were also asked about their access to a smartphone and use of the internet on a smartphone.

#### Sexual Risk Behaviors

This included engagement, during the past 6 months, in anal intercourse with another man; engagement in condomless sex; number of sexual partners; and engagement in group sex, commercial sex, and chemsex. Chemsex is defined as the use of a psychoactive substance (eg, crystal methamphetamine or *batu* or ice, ketamine, ecstasy, and gamma-hydroxybutyrate *G*) before or during anal intercourse [40-42]. The perceived risk of acquiring HIV was measured by a single question, “What do you think is your current risk of getting HIV?” with a dichotomized response of “low” and “high.”

#### Hypothetical mHealth App–Based HIV Prevention Program

After completing the first part of the survey, participants were shown a short video that briefly introduced the hypothetical app-based HIV prevention program:

Imagine you have been asked to participate in a research study! In this study, you will be asked to use an app on your phone for HIV prevention. Advertisements would be placed on social networking apps like Grindr and Hornet to recruit participants into the program. After downloading the app, you will need to create an account, including creating a username and password and entering some personal information, like your name and date of birth. Once you're done, you can use this app for many things! For example, you can order pre-exposure prophylaxis (PrEP) through the app without having to visit a doctor in person. You can communicate with your doctor by sending messages and also receive reminders to take your medications. As part of the research study, you will also be asked to answer a few health-related questions.

Next, participants were shown 4 additional videos illustrating each step involved in the hypothetical app-based program (eg, recruitment, clinical interaction, risk assessment, and weekly reminders), followed by 2-3 questions about ethical concerns (privacy, confidentiality, risks, and benefits) related to each step: for example, (1) recruitment—“Would you be concerned that others might find out about your sexual orientation if you click on the advertisement?”; (2) clinical interaction—“Would you be concerned that doctors and pharmacists receiving your medical information will share it with others?”; (3) risk assessment—“Would you be concerned that research staff might share the health-related information you provide in the app with other people?”; and (4) reminders—“Would you be concerned that other people might see the app messages/reminders on your phone?” In addition, 4 items each examined participants’ overall perceived risks (eg, “I don't think all of my concerns will be addressed without seeing a doctor in person”) and benefits (eg, “I could order PrEP through the app without having to visit a doctor in person”) related to participating in a hypothetical app-based HIV prevention program. All responses to items within each domain (various steps of the hypothetical app program and perceived risks and benefits of participation) were summed to calculate a cumulative scale score with higher values indicating higher levels of concerns or benefits or risks.

### Data Analysis

Analyses were performed using SPSS (version 25.0; IBM Corp). Estimates were evaluated for statistical significance based on 95% CI using the probability criteria of *P*<.05. Descriptive statistics were calculated for all variables. The primary outcome was the willingness to use a smartphone app for HIV prevention. Next, we assessed whether there were demographic differences in the participation choice of MSM for a hypothetical app-based HIV prevention program (those willing vs those who were not willing). We conducted Pearson product–moment correlations between the likelihood that MSM would agree to participate in the hypothetical smartphone app and individual item and composite scale scores. Multivariate logistic regression was used to determine the extent to which cumulative scale scores and separate items together and independently accounted for variability in participation likelihood. The goodness-of-fit of the final multivariate model was assessed using the Hosmer–Lemeshow test [43].

## Results

During the 1-month recruitment period, 1259 participants entered the survey, and 47% (592/1259) consented and completed the screening tool. Of the 592 participants who completed the screening and met the inclusion criteria, 227 (38.3%) did not complete the survey. There were no significant differences between completers and noncompleters for any demographic variables. Of the 365 (365/592, 61.6%) participants who completed the survey, 4 (1%) were eliminated because they failed validation checks (eg, survey duration) and 6 (1.6%) were not included in the analysis because they were identified as female, leaving a final sample of 355.

### Participant Characteristics

Multimedia Appendix 1 provides a summary of the characteristics of 355 participants with a mean age of 33.1 (SD 8.9) years. Most participants were single (238/355, 67%) and university graduates (300/355, 84.5%) and reported not disclosing their sexual orientation to family or friends (224/355, 63.1%). Overall, 22.8% (81/355) of participants had never been tested for HIV, and 42.5% (151/355) reported that they had been tested for HIV at least once in the past 6 months. In terms of sexual behaviors, 76.1% (270/355) of participants reported having anal sex with another man in the past 6 months, with 55.5% (150/270) reporting condomless sex. A minority (42/355, 11.8%) reported having engaged in chemsex, and 16.9% (60/355) of participants reported having engaged in group sex in the past 6 months. Of the 355 participants, 350 (98.6%) reported owning a smartphone and 274 (77.2%) indicated that they use a smartphone “all the time”. Notably, most participants (266/355, 74.9%) indicated their willingness to participate in the hypothetical mHealth app–based HIV prevention program.

### Privacy and Confidentiality Concerns of Using mHealth-Based HIV Prevention Apps

#### App-Based Recruitment

Most participants indicated concerns related to GSN apps (eg, Grindr and Hornet) and their impact on their willingness to participate in mHealth research. Specifically, concerns about others finding out about the participants’ sexual orientation (235/255, 66.2%; *r*=−0.166; *P*=.002) and their HIV status (210/355, 59.2%; *r*=−0.110; *P*=.04) if they clicked on the advertisement were significantly associated with a reduced likelihood of participation. A composite score was created from the items in Table 1 listed under this category (Cronbach *α*=.771; mean 1.9, SD 1.1), which was significantly associated with a reduced likelihood of participation (*r*=−0.152; *P*=.004).

**Table 1:** Survey item frequency and percent and correlation with the willingness to use a smartphone app for HIV prevention.

Variables			Value, n (%)		Correlation
**Concerns related to app-based recruitment**					
	I’d be concerned that others might find out about my sexual orientation if I click on the advertisement.	235 (66.2)		−0.166^a^	
	I’d be concerned that others might find out about my HIV status if I click on the advertisement.	210 (59.2)		−0.110^b^	
	I’d be concerned that my online behavior might be tracked if I click on the advertisement.	260 (73.2)		−0.100	
**Concerns related to app-based clinical interaction**					
	I’d be concerned that doctors and pharmacists receiving my medical information (eg, HIV test results) will share it with others.	226 (63.7)		−0.167^a^	
	I’d be concerned about having medications delivered right to my home.	147 (41.4)		−0.160^a^	
	I’d be worried because I wouldn't be seeing the doctor in person.	167 (47)		−0.171^a^	
**Concerns related to app-based risk assessment**					
	I’d be concerned about providing information about my sexual activities on the app.	201 (56.6)		−0.152^a^	
	I’d be concerned about providing information about my drug use habit on the app.	165 (46.5)		−0.126^b^	
	I’d be concerned that research staff might share the health-related information I provide in the app with other people.	263 (74.1)		−0.120^b^	
**Concerns related to receiving reminders**					
	I’d be concerned that other people might see the app messages/reminders on my phone.	206 (58)		−0.150^a^	
	I’d be concerned about the frequency of the messages/reminders I receive on my phone.	194 (54.6)		−0.070	
	I’d be concerned about the timing of the messages/reminders I receive on my phone.	192 (54.1)		−0.076	
**Benefits of participation**					
	I could order PrEP^c^ through the app without having to visit a doctor in person.	323 (91)		0.068	
	I would get reminder messages to take medications.	323 (91)		0.226^a^	
	I would have access to resources to learn more about HIV prevention.	341 (96.1)		0.117^b^	
	The results of the study could help other individuals like myself.	345 (97.2)		0.137^b^	
**Risks of participation**					
	I don't think I would be comfortable using an app to get medications.	101 (28.5)		−0.139^a^	
	I don't think all of my concerns will be addressed without seeing a doctor in person.	164 (46.2)		−0.181^a^	
	I'm afraid about other people finding out about my participation in the study.	191 (53.8)		−0.249^a^	
	I don't trust researchers to protect information I provide on the app.	169 (47.6)		−0.268^a^	

^a^Correlation is significant at the significance level of 0.001.

^b^Correlation is significant at the significance level of 0.05.

^c^PrEP: pre-exposure prophylaxis.

#### App-Based Clinical Interaction

A significant proportion (226/355, 63.7%) of the participants were concerned about clinical interaction via the app, particularly that the clinicians may share personal health information with others. All the items in this category (eg, clinicians sharing personal health information, home delivery of medications, and not being able to consult with doctors in person) were negatively correlated with participation choice, as was the composite score (Cronbach *α*=.509; mean 1.9, SD 1.5; *r*=−0.234; *P*<.001; Table 1).

#### App-Based Risk Assessment

As illustrated in Table 1, items reflecting concerns related to using an app to assess HIV risk behaviors (sexual: 201/355, 56.6%, *r*=−0.152, *P*=.004; drug use: 165/355, 46.5%, *r*=−0.126, *P*=.02) were negatively correlated with the likelihood of participation in an app-based program. Interestingly, most (263/355, 74.1%) participants were concerned that the research staff might share the health-related information the participants provided via the app with other people outside the research study. The composite score (Cronbach *α*=.710; mean 1.7, SD 1.1) created with these items was significantly correlated with a reduced likelihood of participation (*r*=−0.167; *P*=.002).

#### Receiving Reminders

Just over half of the MSM (206/255, 58%) indicated concerns about other people seeing the messages or reminders on their phones. This was significantly associated with a reduced likelihood of participation (*r*=−0.150; *P*=.005) as was the composite score for these items (Cronbach *α*=.876; mean 1.6, SD 1.3; *r*=−0.110; *P*=.38).

### Benefits of Participation

As shown in Table 1, nearly all participants endorsed the benefit of participating in the hypothetical mHealth app–based HIV prevention program. Of the 355 participants, 323 (90.9%) participants perceived receiving reminder messages to take medications, 341 (96.1%) participants perceived having an app-based platform to learn more about HIV prevention, and 345 (97.2%) participants perceived having altruistic motivation as benefits of participation, all of which were positively correlated with participation. The composite score constructed from these items (*α*=.620; mean 3.7, SD 0.6) also yielded a significant positive correlation with participation choice (*r*=0.198; *P*<.001).

### Risks of Participation

As shown in Table 1, participants reported various perceived risks associated with participation in such a hypothetical app-based study, all of which were negatively correlated with participation. Nearly one-fourth (74/355, 20.8%) of the participants indicated that they would not feel comfortable using an app to get HIV prevention medications. Almost half of the participants (164/355, 46.2%) indicated that their health concerns might not be addressed fully without seeing a health care provider in person. Similarly, 47.6% (169/355) of participants indicated that they did not trust researchers to protect the information they provided on the app. Furthermore, more than half of the participants (191/355, 53.8%) were concerned that other people might find out about their participation in the study. A composite score created from the items in Table 1 listed under this category (*α*=.748; mean 1.7, SD 1.4) was significantly associated with the likelihood of participation (*r*=−0.280; *P*<.001).

### Correlates of Willingness to Use a Smartphone App for HIV Prevention

Table 2 shows the independent correlates and aOR of willingness to use the smartphone app for HIV prevention. In the multivariate logistic regression model, those with a higher degree of perceived benefits (aOR 1.873; 95% CI 1.274-2.755; *P*=.001) were more willing to participate. Conversely, those with increased concerns related to app-based clinical interaction and e-prescription (aOR 0.610; 95% CI 0.445-0.838; 95% CI 0.594-0.899; *P*=.002) and those with a higher degree of perceived risks (aOR 0.731; *P*=.003) were less willing to participate.

**Table 2:** Multivariate logistic regression models of factors associated with willingness to use a smartphone app for HIV prevention.

Variables		Willingness to use the app^a^	
		aOR^b^ (95% CI)	*P* value
Age (years)		0.973 (0.945-1.002)	.07
**Highest education level**			
	Secondary education and below	Reference	—^c^
	Tertiary education (college or university)	1.617 (0.808-3.233)	.17
**Concerns related to participation^d^**			
	App-based recruitment	0.911 (0.682-1.218)	.53
	App-based clinical interaction	0.610 (0.445-0.838)	.002
	App-based risk assessment	0.844 (0.635-1.121)	.24
	Receiving reminders	1.209 (0.947-1.544)	.13
Benefits of participation^c^		1.873 (1.274-2.755)	.001
Risks of participation^c^		0.731 (0.594-0.899)	.003

^a^*R^2^*=0.239; Hosmer–Lemeshow test: *χ^2^*_8,355_=8.0 *P*=.43.

^b^aOR: adjusted odds ratio.

^c^Reference group.

^d^Composite scores.

## Discussion

### Principal Findings

Our findings indicate that mHealth app–based HIV prevention programs are an acceptable research methodology for MSM in Malaysia. This finding is similar to those observed among MSM in other settings [44-49]. Our results also highlight the need to address ethical concerns related to privacy, confidentiality, and data security at all stages of the mHealth research process. These findings are significant in Malaysia, where homosexuality is considered a criminal behavior. Previous research has shown that countries where homosexuality is criminalized often have higher rates of HIV among MSM and lower rates of access to HIV prevention and treatment services compared with their counterparts [50,51]. There are limited physical venues for MSM to meet face-to-face, socialize, and seek health care as homosexuality is illegal owing to the increasingly hostile sociopolitical environment facing LGBT people in Malaysia. With a large proportion of Malaysian MSM using smartphones to seek intimate partners and sexual health information [14], prevention programs need to use innovative platforms that increase the use of anonymous interfaces and reduce in-person interactions.

Although acceptable, our results indicated that Malaysian MSM were concerned about the potential data safety, privacy, and confidentiality concerns and risks and benefits associated with participating in a hypothetical app-based HIV prevention program. Specifically, concerns that information disclosed or shared via the app may be overheard or seen by unintended third parties (eg, colleagues or family members) were noted by more than half of the participants. Confidentiality concerns related to using an app for HIV prevention, including tracking app-based activity or sharing sensitive personal information (eg, health records or engagement in drug and sexual risk behaviors) disclosed via the app were also reported.

Despite these concerns, many participants indicated a willingness to participate, citing improved access to services without interacting with the clinicians in person, receiving personalized reminders, having greater access to information, and having a desire to contribute to a tradition of volunteerism in HIV prevention research [52-54]. These perceived benefits are significantly associated with willingness to participate and are particularly relevant in Malaysia. Previous research has demonstrated that stigma and discrimination are enacted on MSM by health care providers [55-57]. MSM are often hesitant to meet clinicians in person or disclose their sexuality and risk behaviors owing to fear of stigma, discrimination, or criminalization. This hesitation results in extraordinary health disparities and low HIV prevention uptake.

In addition, participants in our sample reported perceived risks associated with participation in mHealth research for HIV prevention, including an inability to fully address health concerns regarding the confidentiality of personal health information provided via the app. The perceived risks related to mHealth research participation negatively influenced willingness to participate. For example, an individual’s perceived negative experiences within technological social spheres may impact their willingness to engage in an mHealth platform, especially for those who feel their sexual orientation or substance use and sexual history could be accessed by, or disclosed without consent to, law enforcement [58,59]. Addressing potential confidentiality, privacy, and data management concerns and relaying these safeguards to participants could increase their willingness to participate. With the increasing use of app-based platforms for HIV prevention services globally, current findings indicate the need to assess participants’ preferences for and feedback to improve engagement in such technological platforms.

### Implications

The findings of this study have significant implications for developing and implementing mHealth app–based programs among MSM in low- and middle-income countries. There is value in using an mHealth app among Malaysian MSM to transform the face of HIV prevention service delivery and personal health management. Leveraging mHealth app–based programs reduces individuals’ discomfort and distrust of disclosing risk behaviors and clinicians’ low cultural competency to work with individuals of diverse sexual identities. Doing so reduces barriers to accessing health care for marginalized populations, such as MSM [60-64], who would have otherwise not opted to seek care. Such an app-based platform would help address issues related to accessibility and use of HIV prevention programs among hard-to-reach and marginalized groups.

The findings highlight many privacy and confidentiality concerns specific to mHealth platforms that need to be addressed to realize their full potential. These concerns become especially relevant in Malaysia and other regions of the world where the burden of social stigma and discrimination is high and where drug use and homosexual behavior are punishable by law. In particular, addressing potential confidentiality and data management concerns before implementing an app-based program and relaying appropriate safeguards to participants are crucial to facilitate widespread public trust and implementation. Such concerns could be addressed by controlling data storage and transmission and accounting for potential third-party data access [27]. The risk of privacy violations can also be minimized by considering how the data are stored locally on the device or in the cloud or transmitted to the research or clinical team. Apps can be placed in secure vaults on mobile devices or can be entirely password-protected with potential secondary authentication measures (eg, biometrics or personal ID number verification) [27,52].

Participants should be informed of the benefits and risks of participating in mHealth research—such as third-party access to data collected via hacking, legal interception (eg, a subpoena), and incidental discovery by someone accessing the phone [27,56]. The developers should maximize opportunities for individuals to control what data they share with the clinical or research team, such as using pop-up messages asking whether they consent to the specific information being transmitted. In addition, controlling the timing of reminders, their form of transmission (such as SMS text messaging vs pop-up message), and coding of messages in a way that only the intended recipient would understand are other ways to prevent a breach in confidentiality. This approach can be tailored according to personal preferences and the participant’s comfort in disclosing health-related information. Furthermore, ensuring data confidentiality through layers of encryption and establishing a secure network connection can prevent remote access to information by potential hackers [52]. Overall, as many of this study’s findings apply to mHealth research among MSM, this study may inform the development and implementation of mHealth app–based HIV prevention programs among key populations in Malaysia and other low- and middle-income countries.

### Limitations

This study had several limitations. First, we used a convenience sample of MSM recruited via gay social media (eg, Grindr and Hornet) rather than a representative sample of all MSM in Malaysia. The web-based methodology in this study may have excluded MSM without access to the internet or those who did not use social networking platforms (eg, Grindr, Hornet, or Facebook) on which the study was advertised. Furthermore, a significant proportion of participants in this study were younger and well-educated, limiting the generalizability of our findings. Second, the use of self-reported measures in this study may have resulted in participants inaccurately reporting socially undesirable behaviors. Third, the use of a cross-sectional study design limited our ability to make causal inferences. Finally, the findings from this study revolved around the features of a hypothetical mHealth app rather than any existing app. Therefore, future research including both MSM (inclusive of both young and older individuals and across the spectrum of education status) and clinical stakeholders is critical. Such research should iteratively solicit feedback at multiple stages of app development to improve acceptability and user experience.

### Conclusions

This study found a high willingness to use a hypothetical app-based HIV prevention program among MSM in Malaysia. The findings further highlighted the role of privacy, confidentiality, and risks and benefits associated with participation in such a program. These findings indicate that privacy and confidentiality concerns related to using an app for HIV prevention efforts are real and critical to ensure widespread public trust and uptake. Therefore, it is important that such platforms are safe and secure. Given the ever-evolving nature of such technological solutions and the complex ethical–legal landscape, the development and implementation of a scientifically and ethically sound app-based HIV prevention program will require early and ongoing engagement with MSM and other relevant stakeholders.

## Appendix

Multimedia Appendix 1 Participant characteristics of Malaysian men who have sex with men, stratified by willingness to use a smartphone app for HIV prevention.

## Abbreviations

aOR: adjusted odds ratio
CS: condomless sex
GSN: geosocial networking
LGBT: lesbian, gay, bisexual, and transgender
MSM: men who have sex with men
PI: principal investigator
