# Supplementary material for: Privacy and Confidentiality Concerns Related to the Use of mHealth Apps for HIV Prevention Efforts Among Malaysian Men Who Have Sex With Men: Cross-sectional Survey Study
Source: JMIR Form Res. 2021 Dec 16;5(12):e28311. doi: 10.2196/28311 (PMC8726055; doi:10.2196/28311)
Supplement: Multimedia Appendix 1 [file formative_v5i12e28311_app1.docx]

**Table 1.** Participant characteristics of Malaysian men who have sex with men, stratified by willingness to use a smartphone app for HIV prevention.

| Variables | | | Total population (N=355) | Willing to use the app^a^ | | *P* value |
| --- | --- | --- | --- | --- | --- | --- |
|  | | |  | Yes | No |  |
|  | | | | | | |
| **Characteristics of participants** | | | | | | |
|  | Age (years), mean (SD) | | 33.1 (8.9) | 32.5 (8.6) | 34.9 (9.5) | .03 |
|  | **Sexual orientation, n (%)** | | | | | .86 |
|  |  | Homosexual or gay or PLU^c^ | 263 (74.1) | 199 (56.1) | 64 (18.0) |  |
|  |  | Bisexual | 77 (21.7) | 56 (15.8) | 21 (5.9) |  |
|  |  | Others^c^ | 15 (4.2) | 11 (3.1) | 4 (1.1) |  |
|  | **Ethnicity: Malay^d^, n (%)** | | | | | .409 |
|  |  | No | 165 (46.5) | 127 (35.7) | 38 (10.7) |  |
|  |  | Yes | 190 (53.5) | 139 (39.2) | 51 (14.4) |  |
|  | **Relationship status, n (%)** | | | | | .487 |
|  |  | Single^e^ | 238 (67.0) | 181 (51.0) | 57 (16.1) |  |
|  |  | Married/partnered | 117 (33.0) | 85 (23.9) | 32 (9.0) |  |
|  | **Highest educational level, n (%)** | | | | | .015 |
|  |  | Secondary education and below | 55 (15.5) | 34 (9.6) | 21 (5.9) |  |
|  |  | Tertiary education (college/university) | 300 (84.5) | 232 (65.4) | 68 (19.2) |  |
|  | **Monthly income, n (%)** | | | | | .977 |
|  |  | ≤3000 MYR**^f^** (USD $750) | 168 (47.3) | 126 (35.5) | 42 (11.8) |  |
|  |  | >3000 (USD $750) | 187 (52.7) | 140 (39.4) | 47 (13.3) |  |
| **Sexual behavior characteristics** | | | | | | |
|  | **Any anal intercourse with another man (last 6 months), n (%)** | | | | | .055 |
|  |  | No | 85 (23.9) | 57 (16.1) | 28 (7.9) |  |
|  |  | Yes | 270 (76.1) | 209 (58.9) | 61 (17.2) |  |
|  | **Condomless anal sex (last 6 months), n (%)^g^** | | | | | .795 |
|  |  | No | 120 (44.5) | 92 (34.1) | 28 (10.4) |  |
|  |  | Yes | 150 (55.5) | 117 (43.3) | 33 (12.2) |  |
|  | Number of male sexual partners (last 6 months), mean (SD) | | 4.5 (6.8) | 4.6 (7.5) | 3.9 (4.1) | .475 |
|  | **Engaged in transactional sex (last 6 months)^h^, n (%)** | | | | | .147 |
|  |  | No | 230 (64.8) | 178 (50.1) | 52 (14.6) |  |
|  |  | Yes | 125 (35.2) | 88 (24.8) | 37 (10.4) |  |
|  | **Engaged in group sex (last 6 months), n (%)** | | | | | .505 |
|  |  | No | 295 (83.1) | 219 (61.7) | 76 (21.4) |  |
|  |  | Yes | 60 (16.9) | 47 (13.2) | 13 (3.7) |  |
|  | **Engaged in chemsex (last 6 months)^i^, n (%)** | | | | | .577 |
|  |  | No | 313 (88.2) | 236 (66.5) | 77 (21.7) |  |
|  |  | Yes | 42 (11.8) | 30 (8.5) | 12 (3.4) |  |
| **Prior HIV testing** | | | | | | |
|  | **Ever been tested for HIV, n (%)** | | | | | .28 |
|  |  | No | 81 (22.8) | 57 (16.1) | 24 (6.8) |  |
|  |  | Yes | 274 (77.2) | 209 (58.9) | 65 (18.3) |  |
|  | **Tested for HIV in the last 6 months, n (%)** | | | | | .055 |
|  |  | No | 204 (57.5) | 143 (10.3) | 61 (17.2) |  |
|  |  | Yes | 151 (42.5) | 123 (34.6) | 28 (7.9) |  |
|  | **Diagnosed with any sexually transmitted infection (past 12 months), n (%)** | | | | | .09 |
|  |  | No | 259 (73.0) | 188 (53.0) | 71 (20.0) |  |
|  |  | Yes | 96 (27.0) | 78 (22.0) | 18 (5.1) |  |
|  | **Current risk of HIV infection, n (%)** | | | | | .315 |
|  |  | Low | 231 (65.1) | 177 (49.9) | 54 (15.1) |  |
|  |  | High | 124 (34.9) | 89 (25.1) | 35 (9.9) |  |
| **Stigma and discrimination** | | | | | | |
|  | **Disclosed sexual orientation, n (%)** | | | | | .33 |
|  |  | No | 224 (63.1) | 164 (46.2) | 60 (16.9) |  |
|  |  | Yes | 131 (36.9) | 102 (28.7) | 29 (8.2) |  |
|  | **Ever felt discriminated by the health care providers due to my sexual identity, n (%)** | | | | | .41 |
|  |  | No | 282 (79.4) | 214 (60.3) | 68 (19.2) |  |
|  |  | Yes | 73 (20.6) | 52 (14.6) | 21 (5.9) |  |

^a^Willingness to use a smartphone app to access HIV prevention services.

^b^PLU: people like us.

^c^Queer, heterosexual, and others.

^d^Includes Indian, Sabahan, Sarawakian, and mixed.

^e^Includes divorced and widowed individuals.

^f^MYR: Malaysian ringgit.

^g^n=270.

^h^Received or provided any things or opportunities (eg, mobile phones, cash, clothes, bags, study, or employment opportunities) in exchange for sex.

^i^Includes crystal meth/*ice*, ketamine, ecstasy, poppers, and GHB/GBL before or during anal sex.
